# Sequences of Zika Virus Genomes from a Pediatric Cohort in Nicaragua

**DOI:** 10.1128/genomeA.00498-18

**Published:** 2018-06-14

**Authors:** Lauren M. Oldfield, Nadia Fedorova, Vinita Puri, Susmita Shrivastava, Paolo Amedeo, Alan Durbin, Iara Rocchi, Torrey Williams, Reed S. Shabman, Gene S. Tan, Angel Balmaseda, Guillermina Kuan, Saira Saborio, Aubree Gordon, Eva Harris, Brett E. Pickett

**Affiliations:** a J. Craig Venter Institute, Rockville, Maryland, USA; b J. Craig Venter Institute, La Jolla, California, USA; c Laboratorio Nacional de Virología, Centro Nacional de Diagnóstico y Referencia, Ministry of Health, Managua, Nicaragua; d Centro de Salud Sócrates Flores Vivas, Ministry of Health, Managua, Nicaragua; e Department of Epidemiology, School of Public Health, University of Michigan, Ann Arbor, Michigan, USA; f Division of Infectious Diseases and Vaccinology, School of Public Health, University of California, Berkeley, Berkeley, California, USA

## Abstract

We report here the whole-genome sequence of 11 Zika virus (ZIKV) samples from six pediatric patients in Nicaragua. Serum samples were collected, and ZIKV was isolated in tissue culture. Both serum and virus isolates were sequenced. The consensus ZIKV genomes are greater than 99% identical to each other.

## GENOME ANNOUNCEMENT

Zika virus (ZIKV) belongs to the Flaviviridae family and can be transmitted to humans via a mosquito vector or other routes, such as sexual transmission. The ZIKV genome is a 10.8-kb single-stranded positive-sense RNA molecule. Although Zika is typically asymptomatic or presents a mild illness, it can cause severe neurological defects, particularly in fetuses of women infected during pregnancy. After the 2007 outbreak in the Federated States of Micronesia (1) and a larger 2013 outbreak in French Polynesia, ZIKV was introduced into Brazil in 2014 and caused a widespread epidemic throughout the Americas (2). This study generated whole-genome sequences for 11 ZIKV samples isolated from a pediatric cohort in Nicaragua.

Serum samples were collected from six pediatric patients between April and July 2016 from Managua, Nicaragua. The patients, aged 2 to 11 years old, were symptomatic and recovered from the infection. Serum samples were collected 1 to 2 days after the onset of symptoms, and the virus was isolated in Aedes albopictus C6/36 mosquito cells. Consensus genome sequence data were generated from the serum samples and the passaged virus isolates. The GenBank accession numbers for five pairs of samples (serum and isolates, respectively, from the same patient) are as follows: KY765318 and KY765321, KY765317 and KY765322, KY765323 and KY765326, KY765325 and KY765327, and KY765319 and KY765320. The sequence from a serum sample from another patient is also reported (KY765324).

The QiaAMP viral RNA mini extraction kit (Qiagen) was used to extract RNA from both serum samples and single-passage virus isolates. RNA sequence-independent single-primer amplification (RNA-SISPA) was performed (3, 4) prior to sequencing on the Illumina MiSeq instrument (2 × 300 bp). Targeted production of cDNA was performed with SuperScript III (Thermo Fisher) using ZIKV-specific primer pairs or random nonamer primers. Amplicons were then generated from the cDNA using ZIKV-specific primers, which are recorded in the GenBank records, and then sequenced using DNA-SISPA on Illumina or Ion Torrent instruments.

Reads were demultiplexed, trimmed, and assembled using the CLCBio *de novo* algorithm. The optimal ZIKV reference genome was identified by using a BLASTn (5) search to query the assembled contigs against our internal set of genomes. Raw reads were then mapped to the selected ZIKV reference genome using a reference-based assembly algorithm within the CLCBio software suite.

In total, over 143,000 reads were assembled for this collection (mean = 13,049 reads; median = 13,025 reads; range = 12,113 to 14,104 reads) with an average coverage of 254×. The coding regions for these assembled genomes, except for the noncontiguous ZIKV/Homo sapiens/NIC/6406_13A1/2016 sequence (GenBank accession number KY765319), were annotated with the VIGOR software (6).

The sequence of each passaged virus was 100% identical to the primary sample from which it was derived. Overall, the sequences in this study had a mean nucleotide identity of 99.76% (range = 99.6% to 100%). This is somewhat expected since these samples were collected from patients within the same geographical and temporal distribution and since there was no selective pressure during single passaging. We expect that the sequence data and associated metadata reported here will provide additional statistical power to future metanalyses.

### Accession number(s).

These sequences reported here were deposited in GenBank under the accession numbers KY765317 to KY765327.
